# Mapping the Metabolic Characteristics and Perturbation of Adult *Casper* Zebrafish by Ambient Mass Spectrometry Imaging

**DOI:** 10.3390/metabo14040204

**Published:** 2024-04-04

**Authors:** Zhi Zhou, Yue Sun, Ji Yang, Zeper Abliz

**Affiliations:** 1 Key Laboratory of Mass Spectrometry Imaging and Metabolomics (Minzu University of China), National Ethnic Affairs Commission, Beijing 100081, China; zhouzhi@muc.edu.cn; 2 College of Life and Environmental Sciences, Minzu University of China, 27 Zhongguancun South Avenue, Beijing 100081, China; 20302095@muc.edu.cn (Y.S.); 23302559@muc.edu.cn (J.Y.); 3 State Key Laboratory of Bioactive Substance and Function of Natural Medicines, Institute of Materia Medica, Chinese Academy of Medical Sciences and Peking Union Medical College, Beijing 100050, China

**Keywords:** ambient mass spectrometry imaging, *casper* zebrafish, spatially resolved metabolomics, whole-body level, metabolic disorder

## Abstract

*Casper*, a type of transparent mutant-line zebrafish, was generated to overcome the opaque trunk of an adult zebrafish for tumor modeling to realize real-time visualization of transplanted cells in vivo. However, the molecular information at the metabolic level has not received much attention. Herein, a spatially resolved metabolomics method based on an airflow-assisted desorption electrospray ionization–mass spectrometry imaging (AFADESI-MSI) system for whole-body zebrafish was used to investigate small molecules and the distribution of adult *casper* (*Mitfa^w2^*^/*w2*^, *roy^a9^*^/*a9*^) and the differences from wild-type zebrafish. Finally, the spatial distribution information of more than 1500 endogenous ions was obtained in positive and negative detection modes, and 186 metabolites belonging to a variety of structural categories were identified or annotated. Compared with wild-type samples, 85 variables, including 37 known metabolites, were screened out. In addition, the disordered metabolic pathways caused by the genetic mutation were excavated, involving downregulation of purine metabolism and arachidonic acid metabolism, upregulation of glycerophospholipid metabolism, and biosynthesis of unsaturated fatty acids. All these results were observed in the most intuitive way through MSI. This study revealed important metabolic characteristics of and perturbation in adult *casper* zebrafish, and provides indispensable fundamental knowledge for tumor research based on it.

## 1. Introduction

A common model organism, zebrafish (*Danio rerio*) has the well-known advantages of being easy to maintain, high fecundity, and juvenile transparency [1]. It has been widely used in disease research due to its high genetic similarity to humans, such as diabetes [2], chronic liver disease [3], nervous system disease [4], etc. Surprisingly, zebrafish has been increasingly taking center stage in cancer research in recent years. Its feasibility for the construction of clinical individualized tumor models has been widely recognized, which offers promise for using zebrafish to tailor specific treatment approaches to individual cancer patients [5].

Due to the opaque trunk of adult zebrafish, both genetic and transplantation models mostly use embryos or larvae, but such an environment cannot simulate the physiological characteristics and pathological process of tumors in adult humans. *Casper*, a transparent mutant line usually lacking light-absorbing melanophores and reflective iridophores, was generated to realize real-time visualization of transplanted cells in vivo. To date, several gene mutants have been generated and applied in tumor research. Tang et al. [6] created *casper*-strain zebrafish with the *rag2^E450fs^* mutant and utilized this to investigate the detailed evolution of metastasis in a subset of *BRAF^V600E^*-driven melanomas. Silja et al. [7] transplanted melanoma cells into transparent *mitfa-BRAF^V600E^*; *p53*^−/−^; *mitfa*^−/−^ fish and quantified metastatic scores at single-cell resolution by combination with computational image analysis. Yan et al. [8] developed an adult *casper* strain *prkdc*^−/−^; *il2rgα*^−/−^ immunocompromised zebrafish, which was able to be used for human cancer cell engraftment robustly excessing 28 days. Staal et al. [9] applied *casper* crossing to *fli-GFP* transgenic fish as recipients of red-labeled human CD34(+) cells to monitor human hematopoietic stem cell transplantation, differentiation, and trafficking in vivo. In a word, the development and application of this strain provide a unique opportunity to uncover the underlying biological processes of cancer in vivo, even at single-cell resolution.

Because constructing an individual tumor model with adult fish is an important developmental path to follow, it is necessary to deeply understand the fish at a molecular level. The genome and proteome of wild-type zebrafish have been fully obtained [10,11,12], as well as the genome and transcriptome of *casper* [13], but a lot of molecular regulatory information is still unspecified. For example, *nacre* is a mutant that carries a mutation in the *mitfa* gene, and how it regulates upstream and downstream molecules is still unclear.

Information on zebrafish metabolomics is also gradually being revealed. Although metabolomic studies based on liquid chromatography–mass spectrometry (LC-MS) have been widely conducted, there are still areas that need elucidation to reveal the spatial information of metabolites at the whole-body level, because most studies have focused on partial organs or partial metabolites [14,15,16]. Airflow-assisted desorption electrospray ionization–mass spectrometry imaging (AFADESI-MSI) technology has been used to develop high-coverage and high-sensitivity visual characterization methods for metabolites [17]. Previously, we established a spatially resolved metabolomic method based on AFADESI-MSI for mapping the whole-body molecular landscape of wild-type zebrafish [18]. In this study, this method was applied to spatially resolved metabolomic research of *casper* zebrafish to understand the distribution of small molecules comprehensively, as well as differences from the wild type, and to explore the correlation between its metabolic phenotype and gene mutations.

## 2. Materials and Methods

### 2.1. Zebrafish and Reagents

Male adult wild-type (AB line) and mutant *casper* (*mitfa^w2^*^/*w2*^; *roy^a9^*^/*a9*^) zebrafish aged 10 months were purchased from the China Zebrafish Resource Center. *Mitfa^w2^*^/*w2*^ is a mutation in the *mitf* gene, completely lacking melanophores. In addition, *roy^a9^*^/*a9*^ is a spontaneously occurring genetic mutation, which has no iridophores, only uniformly pigmented eyes, sparse melanocytes, and translucent skin [19].

Acetonitrile and methanol (HPLC grade) were purchased from Merck (Merck, Darmstadt, Germany). Xylene (analytical reagents) was purchased from Beijing Chemical Industry Group Co., Ltd. (Beijing, China). Purified water was obtained from Wahaha (Hangzhou, China).

### 2.2. Sample Preparation

All zebrafish were starved for 24 h and euthanized in an ice–water mixture. Then, rapid freezing in liquid nitrogen was conducted to fix the fish to a straight, flat state and help to quench biochemical changes in the body. Subsequently, the fish were placed in a 3 × 3 cm^2^ gel freezer mold with water for embedding, and the entire mold was stored at −80 °C. A cryostat (CM1860, Leica Microsystems Ltd., Wetzlar, Germany) kept at −20 °C to −22 °C was used to slice cryosections at the maximum sagittal of a certain thickness (15 μm for positive-ion-mode detection, and 12 μm for negative-ion-mode detection). As ice curls or crumbles more easily during slicing, to reduce the effect of medium breakage, the initial ice slices cut was removed before continuing to slice the sample. Slices for AFADESI-MSI analysis were dried using a vacuum dryer for 6 h, and the adjacent slices were stained with hematoxylin and eosin (H&E).

The tissue homogenate model was prepared by mixing whole-body tissue and saline at a ratio of 1 g/3 mL using a tissue homogenizer. Then, 3 μL of homogenate aliquot of was added to a 3 × 5 mm quasi-rectangular hole, which was formed by the adhesion of the preprocessed polyvinylchloride (PCV) film to a microscope slide. In the same way, it was dried under vacuum conditions for 6 h before performing the analysis. Herein, tissue homogenate models were used as quality control (QC) samples for stability monitoring of the instrumental signal, and analyzed before and after real sample detection every day.

For structure identification, nonpolar and polar metabolites were extracted simultaneously using our previous method of tissue metabolomic research [20]. Briefly, whole-body tissue was cut into pieces, and 410 µL of methanol and 210 µL of water were added. After homogenization at 4 °C, 140 µL of dichloromethane and a mixture of 140 µL of dichloromethane and 210 µL of water were added dropwise and vortexed thoroughly. After standing and centrifuging, the supernatant was the polar extract, which was dried and redissolved in 1.2 mL of an acetonitrile/water (80/20) mixture for LC-MS/MS analysis, and the subnatant was the nonpolar extract, which was dried and redissolved in 120 µL of acetonitrile/water (80/20) mixture for LC-MS/MS analysis. Of course, to improve the efficiency of analysis, extracts from different individuals were blended into a pool sample.

### 2.3. AFADESI-MSI Analysis

Sample detection was conducted using the AFADESI-MSI platform coupled with a Q-OT-qIT hybrid mass spectrometer (Orbitrap Fusion Lumos, Thermo Fisher Scientific, Waltham, MA, USA). Here, a previously established metabolomic method based on AFADESI-MSI applicable to adult zebrafish was adopted, in which the key parameters were optimized [18]. MeOH/H_2_O (8:2, *v*/*v*) was used as the spray solvent with a flow rate of 7 μL/min. The spray voltage and capillary temperature were +3 kV/−3 kV and 350 °C, respectively. The spray pressure was set to 0.6 MPa, and the extracting gas flow rate was 45 L/min. The data were obtained in both positive- and negative-ion modes. Other MS parameters were set as follows: scan range, *m*/*z* 100–1000; automated gain control (AGC) target, 1 × 10^6^; maximum injection time (MIT), 100 ms; resolution, *m*/*z* 200, 120,000. The MSI experiment was conducted at a constant rate of 0.2 mm/s continuously scanning in the *x* direction and a 0.2 mm vertical step in the *y* direction. According to the above scan rate of the mass spectrometer and moving speed of the three-dimensional platform, the spatial resolution of the MSI experiment was about 100 μm.

### 2.4. LC-MS/MS Analysis

The metabolites of interest, i.e., those with distribution characteristics showing differences between groups, were analyzed using LC-MS/MS. Chromatographic separation was performed using an ultrahigh-performance LC system (UltiMate 3000; Thermo Fisher Scientific, Waltham, MA, USA). The nonpolar extract was separated on a reversed-phase Waters HSS T3 column (1.8 μm, 100 mm × 2.1 mm), and the polar extract was separated on a Phenomenex Kinetex HILIC column (2.6 μm, 150 mm × 2.1 mm). The elution method was the same as a previous method [20]. Some key parameters of the MS/MS method were as follows: scan mode, targeted MS^2^; resolution, *m*/*z* 200, 60,000; AGC, 5 × 10^5^; MIT, 200 ms.

### 2.5. Data Processing

The data processing workflow of AFADESI-MSI analysis has been reported in previous studies [21,22,23]. The key points are as follows. MassImager^TM^ version 2.0 (Chemmind Technologies Co., Ltd. (Beijing, China)) was used for ion image reconstruction and background subtraction, and sample normalization was conducted using total ion chromatography. The mass tolerance was set to 0.003 Da, and the subtraction ratio was set to 1. Regions of interest were selected according to their matching H&E images, and the exported .txt files of ion intensities were imported into MarkerView 1.2.1 (SCIEX, Framingham, MA, USA) for peak picking, alignment, and isotope removal.

After using MetNormalizer 1.0 based on support vector regression technology (SVR) for data normalization [24], SIMCA-P (version 14.1) software was used for multivariate statistical analysis. Here, the principal component analysis (PCA) model was used to obtain an overview of the sample distribution and identify outliers. The orthogonal partial least squares discriminant analysis (OPLS-DA) model was used to identify differential metabolites contributing to group clustering.

The structures of the metabolites of interest were analyzed by combining the exact *m*/*z* values, MS/MS spectra, database retrieval, and previous in-house experiences. HMDB (http://hmdb.ca/, accessed on 1 April 2024), METLIN Gen2 (http://metlin.scripps.edu/, accessed on 1 April 2024) and LIPID MAPS (https://www.lipidmaps.org/, accessed on 1 April 2024) were used for database searching. In this study, metabolites that could be matched with MS/MS spectra in the database were denoted as “identified”, and those that could be matched with MS data or predicted based on our previous experience were denoted as “annotated”. The pathway analysis module in MetaboAnalyst 5.0 (https://www.metaboanalyst.ca/, accessed on 1 April 2024) was used for pathway enrichment analysis.

## 3. Results

### 3.1. Data Quality Control

**Figure 1:**
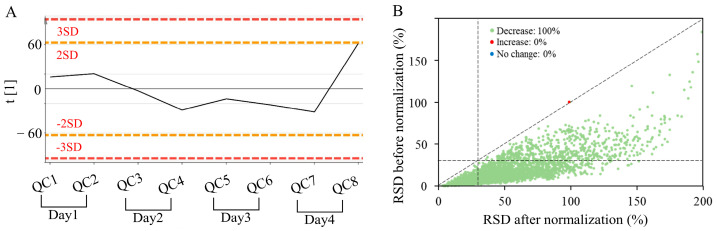
Results of data quality control and normalization obtained by (+) AFADESI-MSI. (**A**) Line plot of QC samples generated by using PCA with one component. (**B**) Comparison plot of the data RSD of QC samples before and after normalization based on SVR. The dashed lines on the x- and y-axis indicate the limit of RSD = 30%.

First, the AFADESI-MSI data in positive- and negative-ion modes of all QC samples were analyzed using PCA for data quality evaluation. Positive-ion-mode detection was conducted for 4 days, and a total of eight QCs were inserted. Negative-ion-mode detection lasted for 2 days, resulting in four QCs. Figure 1A displays a score plot with the principal component number of 1 that originated from MSI data in positive mode. The ion intensity deviations were <2 SD, indicating good stability of the analysis method. Then, SVR-based data normalization was performed to reduce the interday variation to a greater extent. According to Figure 1B, data variation in only one variable in QC samples became larger, and the rest were smaller. Moreover, 80% of the variables had a data deviation of less than 30%, which met the requirements of metabolomic analysis.

### 3.2. Spatial Metabolome of Casper Zebrafish

**Figure 2:**
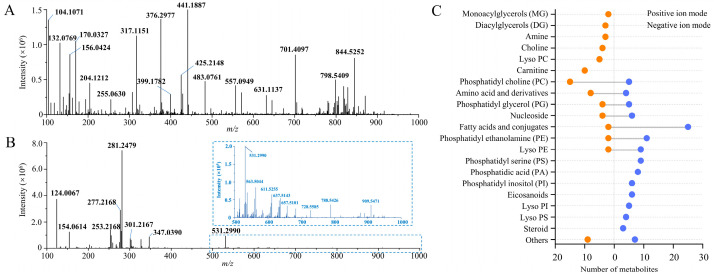
Spatial metabolome of whole-body casper zebrafish obtained by AFADESI-MSI. (**A**) Representative mass spectra in positive-ion mode; (**B**) representative mass spectra in negative-ion mode; (**C**) number of identified or annotated metabolites of different structural categories.

A previously established spatially resolved metabolomic method based on AFADESI-MSI for adult zebrafish was applied to analyze *casper* zebrafish, and the representative mass spectra in both ion modes of the whole-sample region are shown in Figure 2. After peak alignment and isotope elimination, 2163 and 1466 ions were obtained in positive- and negative-ion mode, respectively. This result was basically consistent with the number of ions in wild-type zebrafish obtained earlier. According to image reconstruction using all ions, more than 1500 endogenous ions, that is, distributed in the sample region rather than the background, were obtained in both ion modes.

With reference to the structural identification work basis of previous relevant studies, 75 metabolites in positive- and 117 metabolites in negative-ion mode were annotated or identified, respectively. Tables S1 and S2 in the Supplementary Materials summarize the detailed information on all the representative endogenous metabolites. As illustrated in Figure 2C, more than 20 structural categories of metabolites can be detected in this study, including amino acids, choline, nucleosides, amines, carnitine, fatty acids (FAs) and conjugates, as well as various structural subclasses of lipids, such as monoacylglycerols (MGs), diacylglycerols (DGs), phosphatidylcholine (PC), phosphatidylglycerol, phosphatidylethanolamine (PE), phosphatidylserine (PS), phosphatidylinositol (PI), phosphatidic acid, lyso-PC, lyso-PE, lyso-PS, and lyso-PI. Among them, in positive-ion mode, more metabolites were identified or annotated as PCs, carnitines, and amino acids, with 18, 10, and 8, respectively. In negative-ion mode, most metabolites identified or annotated were FAs, PE, PS, and LPE, with 24, 10, 9, and 9, respectively. The results show the complementarity of the positive and negative detection modes. In short, the majority of the compounds are actually substances in lipid metabolism, especially related to polyunsaturated fatty acids (PUFAs), which is consistent with the biochemical characteristics of fish.

### 3.3. Spatial Metabolome Differentials between Casper and Wild-Type Zebrafish

**Figure 3:**
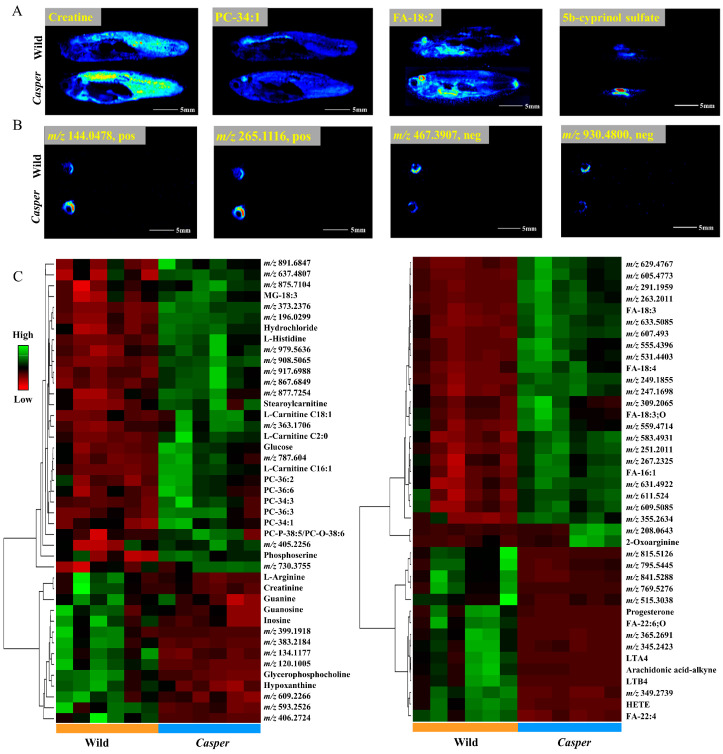
Spatial metabolome differentials between *casper* and wild-type zebrafish obtained by AFADESI-MSI. (**A**) MS images of representative endogenous metabolites; (**B**) MS images of ions distributed specifically in eyes; (**C**) cluster heatmaps of differential variables using (±) AFADESI-MSI data.

First, we selected some representative endogenous metabolites and investigated their spatial distribution in *casper* and wild-type zebrafish. As shown in Figure 3A, the same metabolites had equal distribution. For example, creatine ([M + K]^+^ ion at *m*/*z* 170.0329, pos) was distributed throughout the body, but mainly concentrated in the muscle. PC-34:1 ([M + K]^+^ ion at *m*/*z* 798.5417, pos) showed higher intensity in the brain. FA-18:1 ([M − H]^−^ ion at *m*/*z* 281.2478, neg) showed higher levels expressed in the brain and intestine, and 5b-cyprinol sulfate ([M − H]^−^ ion at *m*/*z* 531.2988, neg) was mainly concentrated in the intestine. Some organ-specific ions previously found in wild-type zebrafish were also detected in *casper* zebrafish with the same distribution characteristics, such as *m*/*z* 144.0478 (identified as 4-methyl-5-thiazoleethanol, pos), *m*/*z* 265.1116 (referred to as thiamine, pos), *m*/*z* 467.3907 (unknown, neg), and *m*/*z* 930.4800 (unknown, neg) all showing specific distribution in the eye region. However, the abundance of the same metabolites in a different-type zebrafish was obviously different.

To explore the differences between them comprehensively, the OPLS-DA model was constructed. After variate filtration, 85 variables with significant intergroup differences were screened out, including 45 in positive mode and 40 in negative mode, and the corresponding cluster heatmaps are provided in Figure 3B. Among the 56 upregulated ions in *casper* zebrafish, 25 variables had two- to fivefold the change factor and 23 variables had more than fivefold. Among the 29 downregulated ions in *casper* zebrafish, 22 variables had a fold change (FC) larger than five. Matching with the known metabolites described above, 25 and 12 variates were annotated in positive- and negative-ion modes, respectively.

### 3.4. Altered Metabolic Pathway Analysis in Casper Zebrafish

**Figure 4:**
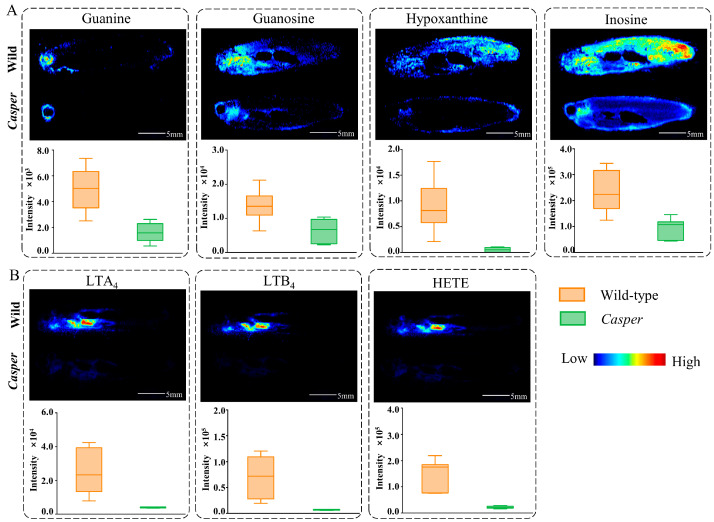
AFADESI-MSI comparison and statistical analysis results of differential metabolites in altered pathways. (**A**) Four metabolites in purine metabolism; (**B**) three metabolites in ARA metabolism.

According to the results of the metabolic pathway enrichment analysis of differential metabolites, several pathways were disturbed in *casper* zebrafish. The top five were purine metabolism, arachidonic acid (ARA) metabolism, glycerol phospholipid metabolism, histidine metabolism, and arginine biosynthesis. Moreover, it should be noted that three or more metabolites were enriched in the ARA metabolism and purine metabolism and only one metabolite was enriched in other pathways. In detail, guanosine, guanine and inosine in the purine metabolic pathway were found to have significant differences in the two types of zebrafish. As shown in Figure 4A, they showed a decreasing trend of expression from the whole-body MS images and data in *casper*. Guanine is an eye-specific metabolite, while the other two metabolites had slightly different distributions in the two strains. In ARA metabolism, LTA_4_, LTB_4_, and hydroxyeicosatetraenoic acid (HETE) had similar distribution in the liver, intestine, and gill, but less distribution in the gill in wild type and more in *casper*. Then, they had an obvious decline in *casper* with a FC more than six. Their spatial distribution and statistical changes are illustrated in Figure 4B.

**Figure 5:**
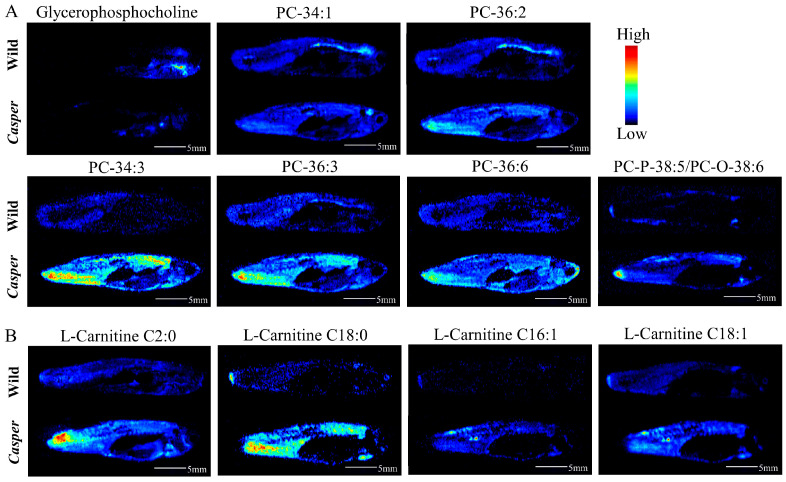
AFADESI-MSI comparison of differential metabolites between *casper* and wild-type zebrafish. (**A**) MS images of seven glycerol PCs; (**B**) MS images of four carnitines.

In addition, we found that there were several PC, carnitine, and unsaturated FA metabolites in the differential list. Generally, as displayed in Figure 5A, PCs were distributed throughout the whole body of zebrafish and were upregulated in *casper*, including PC-34:1, PC-34:3, PC-36:2, PC-36:3, PC-36:6 and PC-P-38:5/PC-O-38:6; however, glycerophosphocholine was downregulated, which is a downstream metabolite of PCs. This may suggest that the expression of enzymes in this pathway was downregulated. Except for abundance, some PCs exhibited different spatial distributions in the two strains of zebrafish, e.g., PC-34:1 was enriched in the brain and spinal cord of wild species, but represented brain enrichment in *casper*. Furthermore, it is interesting to note that the spatial distribution of PCs was more likely correlated with their degree of unsaturation. For example, PC-34:1 and PC-34:3 with the same carbon number and different C=C number had totally different distributions. However, PCs with the same unsaturation number did have similar spatial distribution, such as PC-34:3 and PC-36:3. What is more, it could also be observed from the MSI in Figure 5B that carnitines were also upregulated in *casper*. Similarly, the distribution of these kinds of compounds was correlated with the degree of unsaturation. For example, l-carnitine C18:1 had a different spatial distribution from l-carnitine C18:0 but had very close characteristics of l-carnitine C16:1. The difference in unsaturated FAs, including FA-16:1, FA-18:1, FA-18:3, and FA-18:4, could also be observed visually through MS images.

## 4. Discussion

To our knowledge, this is the first study on the spatial metabolomics of *casper* zebrafish. As expected, we obtained abundant information on endogenous metabolites and their spatial distribution, e.g., more than three neighboring metabolites in the same pathway can be detected simultaneously, which once again supports the reliability and advancement of our previously developed spatially resolved metabolomic analysis method of adult zebrafish. However, it should be emphasized that due to the limitations of the current metabolite identification technology and database, only a small number of metabolites were able to be identified or annotated in this study, i.e., we identified or annotated only 277 ions out of more than 1500 endogenous ions detected, and they came from 186 metabolites. Therefore, the in-depth development of the zebrafish spatial metabolome database or research is very meaningful work. In addition, most of the metabolites identified or annotated in this study belonged to lipid metabolism. Compared with the HMDB database, lipids and their classification record in LIPID MAPS are more comprehensive, but they are not linked with HMDB or MetaboAnalyst. Therefore, the accuracy of pathway analysis is limited and the workload is increased.

**Figure 6:**
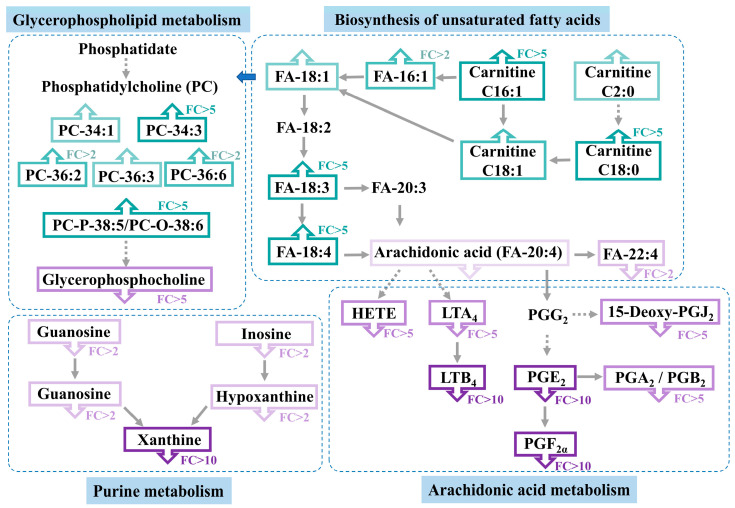
Metabolic disorder in *casper* zebrafish compared with wild-type zebrafish. The green box represents upregulation of metabolites, the purple box represents downregulation of metabolites; the depth of the color is related to the value of FC, and the darker the color, the larger the FC, which divided into FC > 2, FC > 5, and FC > 10.

According to our results, purine metabolism alteration occurred in *casper* zebrafish. Although four differential metabolites were screened out through multivariate statistical analysis, five metabolites were actually detected and all of them were significantly downregulated, as provided in Figure 6. Guanosine and inosine degrade to guanine and hypoxanthine, respectively, both under the action of laccase domain-containing protein 1 (lacc1). Furthermore, guanine and hypoxanthine degrade to xanthine, helped along by guanine deaminase (gda) and xanthine dehydrogenase/oxidase (xdh) severally. Therefore, it is easy to understand that two metabolites directly adjacent to each other in a pathway have similar spatial distribution characters. Moreover, we found that guanosine and inosine also have some similar distribution characteristics, such as a strong response in the gill region. In terms of chemical structure, guanosine has one more –NH_2_ substituent group than inosine, so we speculated that the two metabolites can also be directly converted, although the metabolic reaction between them was not found in KEGG. In addition, xanthine was observed to have specifically high enrichment in the liver, which provides intuitive evidence that purine degradation in zebrafish occurs primarily in the liver.

What is clear is that the color of the fish body is caused by skin pigment cells. One of the most important pigment cells in zebrafish is the iridophore [25], which is found in the skin and scales and mainly contains purine crystals [26,27]. Previous work reported that stacks of guanine crystals with cytoplasm gaps occur within the skin and scales of fish, where guanine crystals act as optical reflectors [28]. Also, hypoxanthine is present in the iridophore of zebrafish skin [29]. Thus, the purines present in these iridophores form the blue stripes and metallic luster of zebrafish. In this study, the distribution of purines in the skin can be clearly observed seen in the MSI. Because the *casper* fish used here has the mutant property of lacking iridophores, purine metabolism is reasonably downregulated. Meanwhile, the iris cells deposit in the retinae of fish, which have a dual function of camouflaging the eye and serving as a light barrier by preventing unfocused light from reaching the retina [30]. It is the high-order organization of multilayered guanine-based crystal reflectors and pigments that facilitates the complex optical response of the iris. Precisely, our results also showed a specific distribution of guanine in the eye region and significant downregulation of it in *casper*.

ARA is an important precursor of eicosanoids that mainly generates downstream leukotrienes (LTs), prostaglandins and HETEs through lipoxygenase, cyclocycase, and cytochrome P450 pathways. Aside from the screened differential metabolites, others were also found to have a decreasing trend in *casper*, as shown in Figure 6. These metabolites have important biological implications for fish. Previous studies have confirmed the correlation between ARA metabolism and pigmentation of the fish body, such as the red *Plectropomus leopardus* exhibiting greater ARA than the black group [31]. Moreover, because prostaglandins and LTs are very important immune factors, the reduction in these substances in *casper* suggests that immune function is weakened, which is precisely one of the advantages of using *casper* for tumor modeling. ARA and its metabolites are also involved in bone formation and remodeling of zebrafish [32], suggesting that the vertebrae of *casper* may be different in size from the wild type.

The upregulation of PCs and carnitines was possible due to the upward biosynthesis of unsaturated FAs. The character of our results seems to be that the same levels of unsaturation have similar metabolite distribution. This phenomenon is most likely by reason of their direct correlation in the metabolic pathway, or it is possible that metabolites with the same unsaturated level have unique biological function, which cannot be confirmed because of the uncertainty of the specific structure of the diacyl group in PCs. PC first loses one fatty acyl group and further loses the second one, which is converted into glycerophosphocholine, which is surprisingly downregulated, and probably because the enzyme expression in this process is inhibited. In any case, we have found some important metabolic disorder in *casper* zebrafish. When this is applied to tumor model construction, biomarkers, and pathogenesis research, these metabolic features should be considered to avoid obtaining unreliable results.

## 5. Conclusions

In this study, *casper* zebrafish with *Mitfa^w2^*^/*w2*^ and *roy^a9^*^/*a9*^ double mutants was first investigated using the spatially resolved metabolomic method based on AFADESI-MSI technology. Abundant information on metabolites and their distribution information in *casper* were obtained, and almost 180 metabolites were identified or annotated. Through multivariate statistical analysis of the spatial metabolome of wild-type zebrafish, 85 variables with significant intergroup differences were screened out and the downregulation of purine metabolism and ARA metabolism revealed, as well as the upregulation of PC and carnitine metabolites. Particularly, we were able to visualize a reduction in purine metabolites in the eyes and skin due to genetic mutations, and also found a potential correlation between the distribution and degree of unsaturation of metabolites containing unsaturated FA chains, such as PCs and carnitines. This study contributes to a more comprehensive understanding of the molecular information on *casper* zebrafish and also provides an important reference for research on disease models based on this mutant.

## Data Availability

The data used and/or analyzed during the current study are available upon request from the corresponding author due to privacy.

## Supplementary Materials

The following supporting information can be downloaded at https://www.mdpi.com/article/10.3390/metabo14040204/s1. Table S1: Representative endogenous metabolites in adult casper zebrafish detected by AFADESI-MSI in positive-ion mode; Table S2: Representative endogenous metabolites in adult casper zebrafish detected by AFADESI-MSI in negative-ion mode. Representative AFADESI-MSI data of casper and wild-type zebrafish under positive- and negative-ion detection modes were uploaded to METASPACE (https://metaspace2020.eu/project/ji-2024 (accessed on 1 April 2024)).
